# Enhanced Electron Heat Conduction in TaS_3_ 1D Metal Wire

**DOI:** 10.3390/ma14164477

**Published:** 2021-08-10

**Authors:** Hojoon Yi, Jaeuk Bahng, Sehwan Park, Dang Xuan Dang, Wonkil Sakong, Seungsu Kang, Byung-wook Ahn, Jungwon Kim, Ki Kang Kim, Jong Tae Lim, Seong Chu Lim

**Affiliations:** 1 Department of Energy Science, Sungkyunkwan University, Suwon 16419, Korea; clainel01@skku.edu (H.Y.); zergpan@skku.edu (S.P.); xuandang@skku.edu (D.X.D.); sk0424@skku.edu (W.S.); rgkang@skku.edu (S.K.); abw890@skku.edu (B.-w.A.); kikangkim@skku.edu (K.K.K.); 2 Department of Smart Fab. Technology, Sungkyunkwan University, Suwon 16419, Korea; qkdwodnr22@skku.edu; 3 Center for Integrated Nanostructure Physics, Institute for Basic Science, Sungkyunkwan University, Suwon 16419, Korea; 4 Institute of Advanced Composite Materials, Korea Institute of Science and Technology, Chudong-ro, Bongdong-eub, Seoul 55324, Korea; jungwon@kist.re.kr; 5 Reality Devices Research Division, Electronics and Telecommunications Research Institute, Daejeon 34129, Korea

**Keywords:** Peierls transition, charge density wave, heat conduction, Wiedemann–Franz law, Lorenz number

## Abstract

The 1D wire TaS_3_ exhibits metallic behavior at room temperature but changes into a semiconductor below the Peierls transition temperature (*T_p_*), near 210 K. Using the 3ω method, we measured the thermal conductivity κ of TaS_3_ as a function of temperature. Electrons dominate the heat conduction of a metal. The Wiedemann–Franz law states that the thermal conductivity κ of a metal is proportional to the electrical conductivity σ with a proportional coefficient of L_0_, known as the Lorenz number—that is, $\kappa =\sigma L_{o}T$. Our characterization of the thermal conductivity of metallic TaS_3_ reveals that, at a given temperature T, the thermal conductivity κ is much higher than the value estimated in the Wiedemann–Franz (W-F) law. The thermal conductivity of metallic TaS_3_ was approximately 12 times larger than predicted by W-F law, implying $L=12L_{0}$. This result implies the possibility of an existing heat conduction path that the Sommerfeld theory cannot account for.

## 1. Introduction

Transition metal trichalcogenide compounds, including NbSe_3_ and TaS_3_, constitute one-dimensional (1D) wires and are metals at room temperature. The first Brillouin zone (BZ) is half-filled, resulting in the first Brillouin zone size quadruple that of the Fermi vector $\kappa_{F}$ [1,2]. However, their structure is unstable. As temperature decreases, the first Brillouin zone of the 1D wire shrinks to a size comparable to 2$\kappa_{F}$. The recrystallization at low temperature, referred to as the Peierls transition, characterizes the gap opening at the Fermi level. In addition, the transition contributes to the periodic modulation of electron density with new crystalline regularity, known as the charge density wave (CDW) [3]. The movement of an electron in a 1D system is strongly correlated with that of other electrons. Any impurity in the 1D system strongly influences the transport behavior so that electrons are isolated by the impurity potential, which is absent from the 2D and 3D crystals [4,5]. The 1D electronic system is a platform for studying superconductivity, metal-insulator transition, strongly correlated phenomena, and new devices [6,7,8,9,10].

In addition to charge transport, the heat transport properties of 1D wires showing CDW are of great interest. Nesting the Fermi vector in the first BZ enhances the interaction between electrons and phonons. Lattice vibrations with large momentum, $\vec{q}=2{\vec{\kappa }}_{F}$, scatter the electrons at the Fermi level. Since electrons (rather than phonons) dominate heat conduction in metals, the carrier relaxation mechanism is important for understanding the heat flow of metallic CDW systems. Recently, in TaS_2_ (a 2D CDW material), the heat conduction between 200 K and 300 K has been significantly suppressed, originating from the strong electron–phonon coupling [11]. Contrary to this, in fresnoite (Ba_2_TiSi_2_O_8_) showing incommensurate CDW (ICDW) material, the quasi-particle of the CDW-phonon produces enhanced heat conduction because of the enhanced group velocity of acoustic phonon [12]. We cannot explain the above-mentioned abnormal heat transport effects from the single viewpoint of an electron or phonon heat conduction because the contribution, role, and detailed mechanism behind CDW in heat conduction in 1D CDW materials are not well understood.

The Wiedemann–Franz (W–F) law assesses the correlation between heat and charge conduction by the electrons in the metal. The electron in this law is assumed to be non-interacting and expected to move in a single band, which is not true all the time, although many metals follow the law well [13]. The electron in the metal has interactions with various entities, including phonons, defects, substrates, and charged impurities. In addition, the Fermi level lies over a few different bands possessing different densities of states and band curvature or effective mass. When these parameters are brought into consideration, the W–F law may fail.

For instance, electrons moving in a 1D structure, exhibiting strong electron-electron interactions, are expected to result in a large *L* [14]. The collective motion of electron and hole plasmons in nondegenerate monolayer graphene violates W-F law with approximately 25 *L/L*_0_ [15]. In 3D bulk materials, multiband transports of both electron and hole carriers in a narrow bandgap semiconductor attribute *L* to far exceed *L*_0_ when the position of the Fermi level inside a bandgap and band curvature is well-optimized.

In this study, we address the behavior of the thermal conductivity of TaS_3_ 1D nanowires above room temperature. The Peierls transition temperature of TaS_3_ is approximately 210 K. Above this temperature, TaS_3_ is a metal. Thus, the heat conduction is dominated by electrons rather than phonons. Our thermal conductivity measurement using the 3ω method addresses the fact that the heat transport of TaS_3_ at room temperature does not obey the W–F law, which assessing the transport of heat and electrical current by electron diffusion across the temperature gradient. The thermal conductivity κ of the metal is expressed as:

(1) $$\kappa =\sigma L_{0}T$$

where *σ* is the electrical conductivity and *L*_0_ is the Lorenz number. In our experiment, the Lorenz number grew as large as 12 times ($L\sim 12L_{0}$). We expected that such a large Lorenz number could result from the strong phonon coupling of the CDW.

## 2. Materials and Methods

### 2.1. TaS_3_ Growth

**Figure 1:**
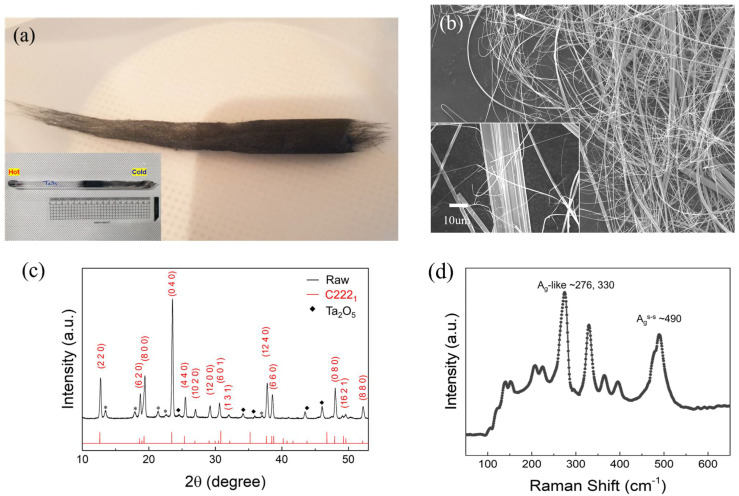
(**a**) Optical image of TaS_3_ grown using chemical vapor transport (CVT); inset shows actual ampoule used for the synthesis of TaS_3_. (**b**) Secondary electron micrograph; inset shows zoom-in image of one of the TaS_3_ bundles. (**c**) X-ray diffraction, and (**d**) Raman spectroscopy of TaS_3_.

Bulk TaS_3_ fiber bundles were grown using the chemical vapor transport method (CVT) [16,17]. We filled a quartz ampoule with tantalum and sulfur powder, a growth precursor, iodine, and a transport agent for synthesis. The Ta and S were weighed carefully to 1:3 in atomic ratio and introduced in the quartz container. We then placed the ampoule inside a two-zone furnace to induce thermal gradient: hot zone (700 °C) and cold zone (500 °C). Figure 1a’s inset shows the ampoule used in the experiment. The synthesis of TaS_3_ fibers lasted for seven days, after which we cooled down the furnace to room temperature for 24 h. Differential heating of our growth conditions contributed to TaS_3_ in different sample geometries. In the cold zone, TaS_3_ grew into fibers, as shown in Figure 1a, with the length of the bulk fiber bundle extending to 10 cm. After breaking the ampoule, we used a small amount of TaS_3_ for further analysis. A scanning electron micrograph in Figure 1b exhibits that the bundle in Figure 1a comprises much narrower bundles of numerous nanowires. Their width was found to vary from a few hundred nm to a few 10 µm as shown in the inset of Figure 1b.

### 2.2. X-ray Diffraction and Raman Spectroscopy

We studied the crystallography of TaS_3_ using an X-ray diffractometer (SmartLab, Rigaku, Japan), which emits Cu Kα radiation (wavelength of X-ray: 1.54059 Å). Since our sample consists of numerous nanowires, we used the Bragg–Brentano mode, which is useful for powder. The X-ray diffraction (XRD) pattern in Figure 1c presents the various crystal planes, which stem from the many nanowires exposed to the incidental X-rays at different crystal directions. The crystal structure of TaS_3_ is orthorhombic with the space group C222_1_, which is supported with reference peaks (red line). The planes of Ta_2_O_5_ are also observed (diamond). Some unknown peaks are indicated with asterisks.

The Raman peaks of TaS_3_ have a strong angle dependence between the E-field direction of the incident laser and the crystal c-axis of TaS_3_; this is responsible for the preferential absorption along the wire [17]. The three prominent peaks of A_g_-like peaks (~276 and ~330 cm^−1^) and an A_g_^s-s^ peak (~498 cm^−1^) with the lower energy side at 276 cm^−1^ and 490 cm^−1^ originating from the o-TaS_3_ phase in Figure 1d are clearly detected, in good agreement with the previous observation [17].

### 2.3. Electrical and Thermal Characterizations of a Bundle of TaS_3_

We performed electrical and thermal conductivity measurements on colinear sample geometry, on which we used Ag epoxy on a single bundle of TaS_3_ to make four contact points. The diameter of the bundle ranges from 15 µm. We softly landed a TaS_3_ bundle on equally spaced small Ag epoxy droplets, which separated the bundle from the sapphire substrate. After connecting the electrical wires, we cured the droplets at 90 °C for 1 h under ambient conditions. We then loaded the substrate into a closed-cycle refrigerator (CCR) (Janis Research Company, Lake Shore, Woburn, MA, USA) to measure the electrical and thermal characteristics. We used the outer two electrodes for the current supply and two inner electrodes for voltage probing. We measured the resistance–temperature characteristics of TaS_3_ by using 6221 DC and AC current sources (Keithley Instruments, Cleveland, OH, USA) and an SR860 lock-in amplifier (Stanford Research, Sunnyvale, CA, USA).

We studied the thermal properties of TaS_3_ using the 3ω method [18]. In this method, AC current *I_o_(f)* at the frequency f=ω/2π is applied up to a level that provokes the Joule heating of our samples. We confirmed the degree of heating of TaS_3_ by checking the voltage at a frequency of 3*f*, $V_{3f}$. Then, the voltage generated at 3*f* because of the Joule heating, and can be expressed as:

(2) $$V_{3f}\sim \frac{2I_{o}^{3}RR^{\prime }l}{\pi^{4}\kappa A\sqrt{{\left(1+4\pi f\gamma \right)}^{2}}}$$

where *I*_0_ is the AC current applied for heating, $R^{\prime }$ is the slope of the R–T curve, *l* is the length, f is the frequency of the applied AC current, γ is the thermal wavelength, and *A* is the cross-sectional area of the TaS_3_ bundle. At the low-frequency limit, $V_{3f}$ is simply expressed as:

(3) $$V_{3f}\sim \frac{2I_{o}^{3}RR^{\prime }l}{\pi^{4}\kappa A}$$

From $V_{3f}$, the thermal conductivity κ can be obtained [19].

To measure the thermoelectric power (TEP), we used dry transfer to deposit a single bundle of TaS_3_ on a SiO_2_/Si (300 nm/500 µm) substrate and patterned electrodes by using e-beam lithography for heating and temperature sensing [20]. We applied a DC current to a serpentine electrode, which causes Joule heating and develops a temperature gradient along the sample. We used a four-terminal method to measure the temperature difference along the sample. Next, we probed the TEP developed over TaS_3_. We used 6221 DC and AC current sources, a 2182 nanovoltmeter, and a 7001 switch system for the characterization [21].

## 3. Results and Discussions

### 3.1. Resistance–Temperature Dependence

**Figure 2:**
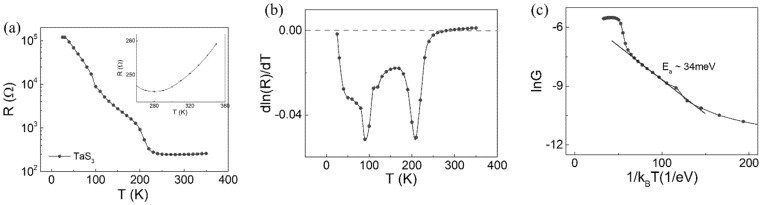
(**a**) Resistance–temperature curve, with an inset zoom-in chart of temperature range between 260 K and 350 K. (**b**) dln(R)/dTvs. T plot, (**c**) $\ln\left(G\right)\mathrm{vs}.\;1/\kappa_{B}T$ plot of TaS_3_ measured using four-point probe method.

As shown in Figure 2a, the resistance–temperature (*R–T*) curve of a bundle of TaS_3_ exhibits a separate slope at approximately 220 K; rapid exponential decay of resistance with multiple slopes occurs in the temperature range of 50–220 K. Such a negative slope in the R–T curve indicates a semiconductor. Then, when the temperature is above 220 K, the inset of Figure 2a shows an up-turn in the *R–T* curve. The inset shows a positive slope in the temperature range of 280–350 K, which is a signature of the metal. The incremental rate of the *R–T* curve is referred to as the temperature coefficient of resistance (TCR), approximately $TCR=5\times 10^{-3}/K$ at room temperature. Electron scattering from optical phonons above room temperature caused the increase in resistance with temperature. The optical phonons such as the A_g_-like mode at 276 cm^−1^ in Figure 1d are expected to be highly populated and scatter the electron above the transition temperature.

To manifest the resistance variation at temperatures below 220 K, we used a derivative of resistance with respect to temperature, as shown in Figure 2b. A sharp dip in the $\frac{dR}{dT}-T$ plot indicates the metal-insulator transition (MIT) of TaS_3_ occurring at 210 K. This characteristic temperature is called the Peierls transition temperature *T_p_*. Before the Peierls transition, the electron density is homogeneous. When the transition occurs, the atomic spacing goes into a modulation. The electrons redistribute themselves with a certain periodicity. Hence, CDW emerges. The CDW of TaS_3_ has been analyzed using various methods at a temperature below *T_p_*, including X-ray diffraction, electron diffraction, and scanning tunneling microscopy. The low-temperature characterization of CDW reports the existence of incommensurate and commensurate CDW depending on the temperature range [22,23,24,25]. Between *T_p_* and 100K, an incommensurate CDW with the wave vector of ${\vec{q}}_{c}=\left(0.252\;\pm \;0.002\right)\vec{c}$ is observed. Below 100 K, the incommensurate CDW turns into commensurate CDW with the wave vector of ${\vec{q}}_{c}=\left(0.250\;\pm \;0.002\right)\vec{c}$. Here, c is the axial direction of TaS_3_. [22]. The wavelength along the axial direction is about 13 Å, whereas the b-axis is nearly 120 Å.

After the transition, the first Brillouin zone size becomes twice the Fermi wave vector; this alludes to the existence of the Fermi surface at the first Brillouin zone edge, which can net q vector connecting the parallel Fermi surface. The atomic restructuring accompanies an energy bandgap at the Fermi level. Although the energy gap varies depending on the temperature, the Arrhenius plot for the conductance *G*,

(4) $$G\sim e^{-\frac{E_{g}}{\kappa_{B}T}}$$

indicates that the bandgap becomes *E_g_* ~ 34 meV in Figure 2c, comparable to a previous result of 67 meV [26]. $\kappa_{B}$ is the Boltzmann constant. For this reason, the resistance increases with decreasing temperature. At lower temperature, the thermal excitation of carriers into the conduction band reduces.

**Figure 3:**
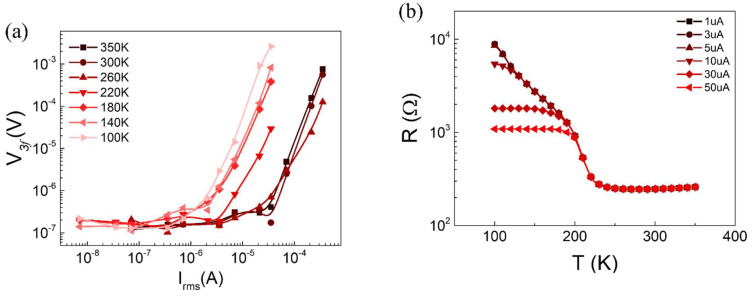
(**a**) *V_3f_*-*I_rms_* curves as a function of temperature. (**b**) Resistance–temperature curves as a function of input current *I_rms_*.

A stiff increase in the resistance below 210 K activated Joule heating under the current used for the resistance measurement of metal TaS_3_. Therefore, we confirmed Joule heating at each temperature by measuring the *V_3f_* voltage, as shown in Figure 3a [18,19]. Depending on the amount of current applied and the sample temperature, the degree of Joule heating varied. For example, applying 10 µA to the sample did not contribute to heating for temperatures down to 260 K, as shown in Figure 3a. However, at temperatures lower than 260 K, noticeable heating occurred, as evidenced by *V_3f_*. As expected from Figure 3a, the *R–T* curve exhibits different slopes depending on the amount of current in Figure 3b. The larger the current, the earlier the *R–T* saturation is found below *T_p_*. After regulating the current at 1 µA, the electric field dependence on the resistance of TaS_3_ observed in previous studies was absent in our case [27,28].

**Figure 4:**
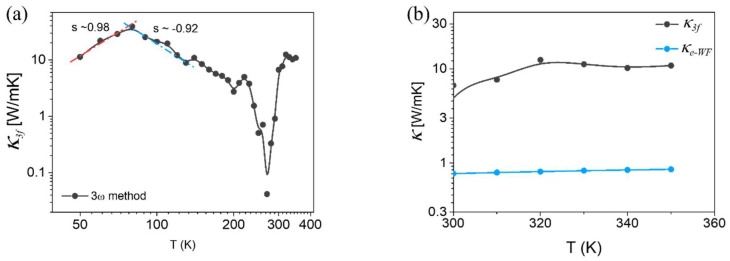

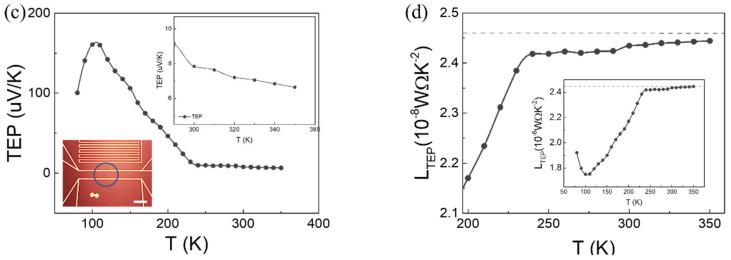
(**a**) Thermal conductivity vs. temperature curve measured using 3ω method. (**b**) Thermal conductivity of TaS_3_ from 3ω method ($\kappa_{3f}$) and electronic thermal conductivity of TaS_3_ from Wiedemann–Franz law ($\kappa_{e-WF}$). (**c**) Thermoelectric power as a function of temperature. Lower-left and upper-right insets show device image and zoom-in plot, respectively. Scale bar measures 20 µm. (**d**) Lorenz number obtained from TEP (L_TEP_) between 200 K and 350 K. Inset is *L_TEP_* from 80 K to 350 K.

The thermal conductivity κ of a bundle of TaS_3_ as a function of temperature is shown in Figure 4a. The *R–T* curve in Figure 2a shows that, with temperatures between 50 and 200 K, TaS_3_ is a semiconductor. For semiconductors, lattice vibrations are the dominant heat carriers. In the log (κ)–log(*T*) plot in Figure 4a, the heat conductivity of our samples is characterized by the peak appearing at approximately 80 K. The Debye temperature (*D_T_*) of TaS_3_ is lower than 130 K [29]. At temperatures well below *D_T_*, an increase in temperature promotes the heat capacity *C_v_* of the lattice, $C_{\nu }\sim T^{3}$, for bulk materials, whereas the phonon mean free path is constant. Thus, the cube dependence on the thermal conductivity of the lattice $\kappa_{p}$ on *T^3^* is expected when *T* << *D_T_*. In contrast to this expectation for our TaS_3_ bundle, Figure 4a shows that the red line—our fit-to-log (κ)–log(*T^β^*) plot at a temperature below 70 K—reveals an exponent, *β* ~ 0.98. This result implies that our TaS_3_ bundle is likely a 1D heat conductor. Although individual TaS_3_ nanowires are expected to be physically entangled in a bundle for heat conduction, *β* ~ 0.98 supports the expectation that nanowire-to-nanowire interaction should be quite low. At *T* >> *D_T_*, the heat capacity saturates, and the phonon mean free path $\lambda_{p}$ is inversely proportional to the temperature, $\lambda_{p}\;\sim \;1/T$. The thermal conductivity of the phonon, $\kappa_{p}$, decreased with increasing temperature. As predicted, the blue line—our fit-to-the-curve plot in Figure 4a—shows an exponent *β* ~ −0.92 in the temperature range from peak temperature to 150 K, implying that the behavior of phonon-driven heat conduction is well-presented.

However, above *T_p_*, the thermal transport behavior deviates significantly from the *1/T* behavior. Specifically, it fluctuated significantly around *T_p_*. The extraction of κ from Equation (3) requires the slope of the *R–T* curve, as shown in Figure 2a. In this temperature range, estimating κ is difficult because of the extremely low slope of *R-T* curve at the transition between the semiconductor and metal. The dependence of κ on the temperature becomes proportional when the temperature increases above 280 K. Figure 2a indicates that TaS_3_ is a metal above 280 K. In metals, heat is carried by electrons. The thermal conductivity of an electron is described as:

(5) $$\kappa_{e}=\frac{1}{3}C_{\nu }\nu_{F}\lambda$$

where $C_{\nu }$ is the heat capacity, $\nu_{F}$ is the Fermi velocity, and λ is the mean free path of the electron [30]. The electron heat capacity is proportional to *T*, as in $C_{\nu }\sim T$. λ is inversely proportional to T, as in λ~1/T, because of the increasing phonon scattering. Since E_F_ changes negligibly within a narrow temperature range between 300 K and 350 K, we assumed the overall trend of $\kappa_{3f}$ to be constant. In contrast to this general assumption, $\kappa_{3f}$ in this temperature range shows a proportional increase in the temperature, as shown in Figure 4b.

When heat conduction occurs in the metal wire, the electrons diffusing from the hot to cold region transport the heat current as well as the charge current, whose relation is expressed in Equation (1). In the equation, *L*_0_ is further expressed as:

(6) $$L_{o}=\frac{\pi^{2}}{3}{\left(\frac{\kappa_{B}}{q}\right)}^{2}=2.44\times 10^{-8}{\;W\Omega K}^{-2}$$

where *q* is the electrical charge. With this relation, we can evaluate the thermal conductivity from the electrical conductivity *σ*. The electron thermal conductivity based on the Wiedemann–Franz law,$\;\kappa_{e-WF}$, in Figure 4b scales with temperature. However, Figure 4b shows a large discrepancy between $\kappa_{e-WF}$ and $\kappa_{3f}$.

In certain cases, a different mechanism incorporates electron scattering for heat and charge conduction. For instance, at very low (high) temperatures, impurities (optical phonons) solely hinder both charge and heat conduction by electrons. In this case, *L* approaches the theoretical value, *L*_0_. However, at moderate temperatures, the optical phonon and impurity scattering may contribute differently to heat and charge transport, respectively. The relaxation time approximation (RTA) model differentiates the scattering events of thermally and electrically excited electrons. Therefore, $L_{o}=\frac{\pi^{2}}{3}{\left(\frac{\kappa_{B}}{q}\right)}^{2}$ commonly loses its validity in such cases. In the RTA model, the ratio is further expressed as:

(7) $$\frac{\kappa }{\sigma }=\frac{\tau_{\kappa }}{\tau_{\sigma }}L_{o}T$$

where $\tau_{\kappa }$ and $\tau_{\sigma }$ are the scattering times for heat and charge transport [31]. Since measuring $\tau_{\kappa }$ and $\tau_{\sigma }$ is difficult, Snyder et al. proposed an empirical law [32] that enables us to estimate the Lorenz number by measuring the thermoelectric power (TEP), expressed as:

(8) $$L=1.5+exp\left(-\frac{\left|S\right|}{116}\right)$$

This expression holds for a single parabolic band and acoustic phonon scattering. Here, *S* is the Seebeck coefficient.

We scrutinized the TEP (*S*) of TaS_3_ to estimate *L* for the metallic one. The lower-left inset in Figure 4c shows the device, with TaS_3_ located inside a circle between the electrodes. Below 210 K, the TEP increases with decreasing temperature. The Mott relation, S~1/G, can explain the increase in TEP at low temperatures, with G as the electrical conductance [33]. The TEP near room temperature ranges around 10 µV/K, indicating the hole as a majority carrier, and remains almost unchanged down to 250 K, as shown in the upper-right inset of Figure 4c. This attribute causes an empirical L to remain almost constant in the temperature range between 250 K and 320 K, as shown in Figure 4d. We observed L_TEP_ from the TEP measurement: $L_{TEP}=2.42\times 10^{-8}\;{W\Omega K}^{-2}$, which is only a small percentage lower than *L*_0_. When *T* < 250 K, the *L_TEP_* decreased significantly. At low temperatures, TaS_3_ opens a small bandgap. In nondegenerate narrow-gap semiconductors, carriers from both the conduction and valence bands participate in the heat conduction. Both holes and electrons diffuse in the same direction against the temperature gradient. In the case of the multiband transport without interband interactions, the total L varies depending on the position of the Fermi level inside the bandgap, $\frac{E_{F}}{\kappa_{B}T}$, and the band curvature. Thus, it is expressed as a linear combination of *L* from each band, which is weighted by the partial electrical conductance. In other words,

(9) $$L=\frac{\sum_{i}G_{i}L_{i}}{\sum_{i}G_{i}}$$

$G_{i}$ indicates the conductance of the *i*th band. Hence, *L* varies widely [34]. In the inset of Figure 4d, *L_TEP_* converges to $L_{TEP}=1.5\times 10^{-8}\;{W\Omega K}^{-2}$ as the temperature decreases.

### 3.2. Characterization of Temperature-Dependent Lorenz Number

**Figure 5:**
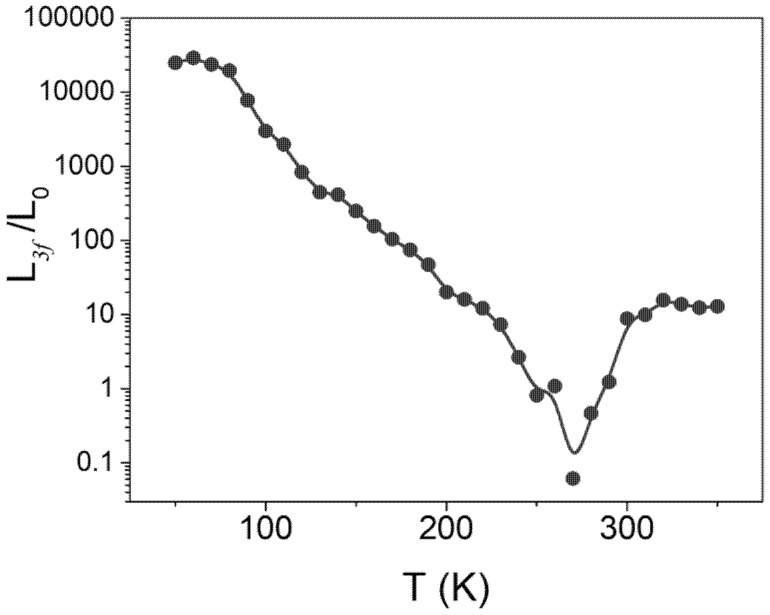
$L_{3f}/L_{0}$ vs. T in temperature range between 50 K and 350 K.

Since our 3ω method allows us to characterize thermal and electrical conductivity simultaneously as a function of temperature, we could also extract L_3*f*_ and classify the electronic and phononic heat conduction. As stated above, this approach may not hold at temperatures below *T_p_* because TaS_3_ is a semiconductor. This ratio is expected to provide a qualitative understanding of heat conduction by electrons only above room temperature. Figure 5 shows the ratio of *L_3f_* to *L_0_, L_3f_*/*L*_0_ obtained from the 3ω method. The curve of *L_3f_/L*_0_ below 200 K, at which TaS_3_ is a gapped semiconductor, changes remarkably. In addition, the ratio grows much larger than the unity, ranging from 10 to 10,000. In our measurements, the thermal conductivity in this temperature range results from lattice vibrations with a large value of *L_3f_/L*_0_, far above unity, as shown in Figure 5. In contrast to the low-temperature behavior, the curve of *L_3f_/L*_0_ above 300 K does not change remarkably. The metallic TaS_3_ maintains a ratio much larger than unity, approximately, $L_{3f}/L_{0}=12$ and gradually increases with temperature. The material properties of TaS_3_ above and below *T_p_* are compared in Table 1. 

Our results contrast with previous studies from the perspective that most Wiedemann–Franz law violations show that *L*_3*f*_*/L*_0_ is slightly smaller than unity [35]. In addition, they observed violations at low temperatures [15]. Specifically, a large *L*_3*f*_*/L*_0_ exceeding 1000 is theoretically predicted for 1D metal wires exhibiting Luttinger liquid behavior [9,14]. TaS_3_ is a 1D metal near room temperature, as shown in Figure 1a. However, the Luttinger liquid behavior from TaS_3_ is expected to prevail below 100 K [5,36]. Therefore, the large violation of the Wiedemann–Franz law does not stem from the 1D electronic nature.

The collective heat transport of quasiparticles can explain another possibility for the breakdown of the Wiedemann–Franz law. Coupled lattice vibrations with charge density waves, referred to as phasons, are identified as another pathway for heat conduction in addition to electrons and phonons, respectively [8,12,37]. *L*_3*f*_*/L*_0_ in Figure 4a,b, indicate that the conventional heat carriers, phonons, and electrons in metal TaS_3_ provide only marginal contributions. CDW-phonon coupling is expected to dominate the thermal transport. Thus, the thermal conductivity should be expressed as $\kappa_{total}\approx \kappa_{CDW-ph}$. The CDW manifests itself strongly at low temperatures. However, when the temperature surpasses the transition temperature, the periodicity of CDW becomes weak. For instance, from an STM study conducted at room temperature, a weak sign of CDW was observed [25]. Furthermore, above *T_p_*, the possibility of forming a pseudogap was proposed. For this reason, in our temperature range, we propose that the CDW is vivid and participating in heat conduction above *T_p_*.

## 4. Conclusions

The flow of heat and charge in 1D structure contains exciting and exotic sciences compared with their bulk counterparts. For instance, the charge transport in a 1D wire exhibits a much stronger interaction, referred to as the Luttinger liquid. As a result, the charge and spin propagate independently. The thermal property in 1D, in the case of carbon nanotubes (CNTs), displays dimensional dependence. TaS_3_ is a 1D metal wire above *T_p_*, which shows unprecedented heat transfer properties, as stated in this paper. In order to achieve significant advancement in the understanding of heat conduction in 1D electronic systems, more scrutiny is needed of the interaction between CDW and lattice vibrations, the alteration of the phonon band and dispersion, and the contribution of the quasiparticle to heat conduction.

## Figures and Tables

**Table 1:** Whole measurement results from single TaS_3_ fiber.

	*R* [Ω]	TCR [×10^−3^/K]	$\kappa_{3f}$ [W/mK]	$\kappa_{e-WF}$ [W/mK]	TEP [μV/K]	$L_{3f}/L_{0}$
Metallic phase (300 K)	246.37	5.53	6.68	0.86	7.84	8.80
CDW phase (100 K)	8859.47	−57.76	21.06	7.16 × 10^−3^	160.85	2991.68

## Data Availability

The data presented in the article will be shared on request by the corresponding author.

## Nomenclature

Symbol	Description	Unit
${\vec{\kappa }}_{F}$	Fermi wave vector	1/m
$\vec{q}$	Momentum vector	kg·m/s
*Σ*	Electrical conductivity	S/m
*ω*	Angular frequency	rad/s
κ	Thermal conductivity	$\frac{W}{\mathrm{mK}}$
$\kappa_{e}$	Electron thermal conductivity	$\frac{W}{\mathrm{mK}}$
$\kappa_{p}$	Phonon thermal conductivity	$\frac{W}{\mathrm{mK}}$
$\kappa_{3f}$	3f thermal conductivity	$\frac{W}{\mathrm{mK}}$
$\kappa_{CDW-ph}$	CDW-phonon coupled thermal conductivity	$\frac{W}{\mathrm{mK}}$
$\kappa_{e-WF}$	Thermal conductivity from σ and Wiedemann–Franz law	$\frac{W}{\mathrm{mK}}$
$\kappa_{total}$	Total thermal conductivity	$\frac{W}{\mathrm{mK}}$
$L_{0}$	Theoretical Lorenz number	$10^{-8}\;WΩK^{-2}$
$L_{TEP}$	Lorenz number from TEP	$10^{-8}\;WΩK^{-2}$
$L_{3f}$	Lorenz number from 3f	$10^{-8}\;WΩK^{-2}$
f	Frequency	Hz
$I_{0}$	AC current (RMS)	A
$V_{3f}$	3*f* voltage	V
*R*	Electrical resistance	Ω
*R′*	Slope from resistance-temperature curve	$\frac{Ω}{K}$
*A*	Cross-sectional area	m^2^
l	Sample length	m
γ	Thermal wavelength	m
*T*	Temperature	K
*T_p_*	Peierls transition temperature	K
$\kappa_{B}$	Boltzmann constant	eV/K
*G*	Electrical conductance	S
$D_{T}$	Debye temperature	K
$\lambda_{p}$	Phonon mean free path	m
λ	Electron mean free path	m
$C_{v}$	Specific heat capacity	J/g/K
$v_{F}$	Fermi velocity	m/s
q	Elementary electric charge	C
$\tau_{\kappa }$	Scattering time of heat transport	s
$\tau_{\sigma }$	Scattering time of charge transport	s
S	Thermoelectric power	$\frac{\mu V}{K}$
$E_{F}$	Fermi level energy	eV
